# Status of Respectful Maternity Care Among Women Availing Delivery Services at a Tertiary Care Center in Central India: A Cross-Sectional Study

**DOI:** 10.7759/cureus.27115

**Published:** 2022-07-21

**Authors:** Sarita K Sharma, Pragati G Rathod, Kanchan B Tembhurne, Ujwala U Ukey, Uday W Narlawar

**Affiliations:** 1 Community Medicine, Government Medical College and Hospital, Nagpur, Nagpur, IND

**Keywords:** tertiary care centre, cross sectional, women, quantitative, abuse, disrespect, respectful maternity care

## Abstract

Introduction

There are numerous reports of disrespectful, abusive, or neglectful treatment during childbirth from health facilities worldwide. Although India has substantially increased the number of hospital deliveries and reduced the maternal mortality ratio, the quality of intrapartum and immediate postpartum care for delivering mothers has not been given much importance. Therefore, assessing mistreatment and quality of care during childbirth is vital for promoting respectful maternity care.

Methods

A descriptive hospital-based cross-sectional study was carried out in a tertiary care center in central India. A convenience sampling method was used, and a total of 150 consecutive consenting women aged 18-49 years who delivered in the study setting were included. Data was collected using a predesigned and pretested questionnaire based on seven major categories per the Respectful Maternity Care (RMC) Charter.

Results

All the 150 women in the present study, i.e., 100%, experienced at least one form of disrespect during their labor, childbirth, or postnatal period at the hospital. The mean scores for domains of non-confidential care (0.59), non-consented care (0.95), abandonment or denial of care (1.21), and physical abuse (1.26) are low.

Conclusion

The findings of the present study shed important light on the current state of respectful maternity care in the study area. Though mothers are noticing and reporting positive changes in maternity care practices, respectful maternity care still has a long way to go.

## Introduction

The remarkable progress in the field of health care services in recent decades has resulted in a considerable drop in maternal and neonatal morbidity and mortality rates worldwide. With women being increasingly incentivized to utilize facilities for childbirth, institutional delivery rates have improved sizeably. However, many women across the globe still experience disrespectful, abusive, or neglectful treatment in health institutions during childbirth [1,2,3]. This certainly indicates that there is yet a long way to go in making maternity care more respectful and dignified. Women seeking maternity services often encounter rude and uncourteous treatment at the health care facilities in India [4]. Disrespectful and abusive treatment during childbirth includes outright physical abuse, profound humiliation, verbal abuse, unconsented medical procedures, lack of confidentiality, failure to get informed consent, and refusal to give pain medications. Gross violations of privacy, refusal of admission to health facilities, neglecting women during childbirth leading to life-threatening complications that are avoidable, and detention of women and their newborns in facilities due to an inability to pay also amount to disrespect and abuse [5,6]. Lack of respect for women in childbirth facilities can be a significant deterrent to utilizing maternity care services. The relationship between pregnant women and maternity service providers is thus crucial as experiences with caregivers during pregnancy and childbirth may positively or negatively impact women. This experience may either comfort them or cause emotional trauma and thus support or undermine their confidence and self-esteem [7].

While many governments, professional societies, researchers, international organizations, civil society groups, and communities worldwide have already highlighted the need to address this problem, in many instances, policies to promote respectful maternal care have not been adopted, are not specific or have not yet been translated into meaningful action [1,7]. In 2010, in a landscape report, Bowser and Hill described seven major categories of disrespect and abuse that childbearing women encounter in facility-based maternity care. These categories overlap and occur along a continuum from subtle disrespect and humiliation to overt violence [6, 7]. In 2011, White Ribbon Alliance published the Respectful Maternity Care Charter: The Universal Rights of Childbearing Women, a document utilized in many countries as an advocacy and program tool [7]. The WHO released a statement in 2015 reasserting the fundamental human rights of women in childbirth [1]. Ministry of Health and Family Welfare, Government of India, launched the ‘LaQshya’ initiative in 2017, which is intended to improve the quality of care in labor rooms and maternity operation theatres. Under this, states are urged to undertake concerted efforts to ensure that respectful and high-quality maternal care is provided to each woman during delivery and immediate postpartum [4]. Since then, the field of respectful maternity care has continued to grow with increased focus.

With this background and keeping in mind the dearth of literature on this topic, mainly from Central India, the present study was conducted to assess the current status of respectful maternity care in a tertiary care center in central India. This article was presented as a scientific paper at the National IAPSM conference on March 4, 2022.

## Materials and methods

Study design, setting, and duration

A hospital-based descriptive cross-sectional study was carried out in the postnatal wards of the Department of Obstetrics and Gynecology of a tertiary care center in Central India for six months, from October 2021 to March 2022. The study setting is a public health institute, wherein an average of 20 to 30 deliveries are conducted per day.

Study population (inclusion and exclusion criteria)

Women aged 18-49 years who delivered in the tertiary care center in Central India constituted the study subjects. Women having adverse obstetric outcomes like stillbirth or neonatal death and thus not in a state to respond, and those who were not willing to participate were excluded from the study.

Sample size

Considering the prevalence of respectful and abuse-free maternity care to be 57%, as per the study done by Wassihun B and Zeleke S [8], the required minimum sample size was calculated to be 147 by taking desired confidence limit (1-α) as 95% and the absolute precision (d) as 8%. However, the actual sample taken was 150.

Sampling technique

A convenience sampling method was used. Postpartum women from the maternity wards who met the inclusion criteria were invited to participate in the study. Thus consecutive consenting women were included in the study till the desired sample size was achieved.

Ethical considerations

The Institutional Ethics Committee (IEC) of Government Medical College, Nagpur, reviewed and approved the study protocol vide letter no 1306/EC/Pharmac/GMC/Nagpur. Written informed consent in the subject's vernacular language was taken before enrolment for study after apprising her about the nature and purpose of the study. The participants were assured of complete privacy, confidentiality, and anonymity.

Data collection method

The participants were briefed on the objectives and methods of the study and were requested to sign a written informed consent in case they were willing to participate in the study. A face-to-face interview using a predesigned and pretested questionnaire was carried out on the day of discharge.

Data collection tool

The study tool comprised of a questionnaire consisting of two parts. The first part of the questionnaire included information on participants' sociodemographic characteristics such as age, residence, religion, marital status, education, occupation, type of family, and socioeconomic status. The second part comprised of questions based on seven major categories of the Respectful Maternity Care (RMC) Charter [7], which included - physical abuse, non-consented care, non-confidential care, non-dignified care, discrimination based on specific attributes, abandonment or denial of care, and detention in a facility.

The English version of the questionnaire was translated into vernacular languages (Marathi and Hindi) to ensure content clarity for participants. The Marathi and Hindi versions were transcribed to English to check for consistency. For respectful maternity care, all the items in each of the seven major domains were scored on a 3-point Likert scale - always, sometimes, never as 2, 1, 0, respectively, or yes, no as 2, 0, respectively. For some of the items (like, Did the staff scream or shout over you?), reverse coding was done wherein always, sometimes, never were scored as 0, 1, 2, respectively, or yes, no scored as 0, 2, respectively. The highest attainable score was 2, and the higher the score better was the quality of respectful maternity care.

The questionnaire was pretested by conducting a pilot study on 5% of the sample size (eight respondents) to identify any ambiguity and check for consistency and acceptability of the questionnaire. Necessary modifications were made to the questionnaire accordingly. The filled questionnaires were collected and checked for consistency and completeness daily by the principal investigator.

Data analysis

Data were entered in Microsoft Excel software and were analyzed using Epi Info version 7.2.2.5 (CDC, Atlanta, Georgia) software [9]. The data were expressed in terms of percentage, mean, and SD.

## Results

Sociodemographic characteristics of respondents

The total number of study subjects enrolled was 150, of which 47 (31%) had undergone cesarean section and 103 (69%) had a normal delivery. Apart from these 150 mothers, two had adverse obstetric outcomes and were not in an emotional state to participate; hence were excluded from the study. The majority of the study subjects, 147 (98%), were 18-35 years old. The mean age of participants was 25.75, with an SD of 4.28 years. The minimum reported age of respondents was 19 years, and the maximum age was 40 years. All the study subjects were married. Most of the respondents, 83 (55.33%), were from rural areas. Other sociodemographic characteristics of the study subjects are shown in Table 1. 

**Table 1:** Sociodemographic profile of study subjects.

Variable	Categories	Study Subjects	
		No.	%
Age (in years)	18-25	84	56
	26-29	37	24.67
	>30	29	19.33
Religion	Hindu	118	78.67
	Buddha	26	17.33
	Others	6	4
Educational Status	Graduate/Post Graduate	51	34
	Diploma	51	34
	High School and Below	48	32
Occupational Status	Skilled	5	3.33
	Semi-Skilled	10	6.67
	Unskilled	26	17.33
	Homemaker	109	72.67
Type of Family	Nuclear	49	32.67
	Joint	42	28
	Three Generation	59	39.33
Socio Economic Status (B. G.Prasad Scale)	Class I (Upper Class)	5	3.33
	Class II (Upper Middle Class)	28	18.67
	Class III (Middle Class)	40	26.67
	Class IV (Lower Middle Class)	44	29.33
	Class V (Lower Class)	33	22

The domains of respectful maternity care

Physical Abuse

In the domain of physical abuse of respectful maternity care (Table 2), most of the respondents, 103 (68.67%), reported that they were not provided physical comfort during the intranatal period. More than half of females, i.e., 82 (54.67%), reported that health care providers used some form of physical force or abuse, including pushing, slapping, pinching, or any gesture towards slapping or hitting during examination and delivery. Hundred and forty-five (96.67%) and 142 (94.67%) females reported that the health care providers never touched or examined them and their babies inappropriately or with a lack of care. Denial of fluid, provision of physical comfort, and use of physical force or abusive behavior, were found to be the lowest scoring items with a mean score of 0.36, 0.62, and 0.91, respectively. The overall mean score for the domain of physical abuse was 1.26.

**Table 2:** Physical abuse domain. *: Reverse-coded items.

Sr. No.	Physical Abuse	Yes	No	Mean Score
		No. (%)	No. (%)	Mean (SD)
1	Was physical comfort (e.g., raising the head-end of the table, giving pillows, etc.?)	47 (31.33)	103 (68.67)	0.62 (0.93)
2*	Was fluid denied to you during delivery? (n=78) (25 did not ask for fluid and 47 had undergone lower segment cesarean section[LSCS])	64 (82.05)	14 (17.95)	0.36 (0.77)
3	Did the care providers give pain relief medication? (n=96) (47 had undergone LSCS, no episiotomy was given in 7)	139 (92.67)	11 (07.33)	1.85 (0.53)
4*	Did they use any physical force or abusive behavior with you (during the examination, delivery, stitches)?	82 (54.67)	68 (45.33)	0.91 (1.00)
5*	a) Did the care providers touch /examine you inappropriately or with a lack of care?	5 (3.33)	145 (96.67)	1.93 (0.36)
	b) Did the care providers touch /examine your baby inappropriately or with a lack of care?	8 (5.33)	142 (94.67)	1.89 (0.45)

Non-Consented Care

Non-consented care in study subjects is depicted in Table 3. All the study participants reported that the service providers never introduced themselves. Though 128 (85.33%) females were allowed a birth companion of their choice to accompany them in wards, 102 (99.03%) females were not allowed to have a birth companion in labor rooms. Also, 81 (54%) females stated that verbal consent was taken only sometimes, whereas 39 (26%) reported that the staff never took verbal consent before any action or procedure. None of the females were allowed to deliver in the position of their choice. It was heartening to note that the service providers never separated the study subjects from their babies without their consent. In this domain, the mean score for items related to health care providers introducing themselves and allowing the study subjects to deliver in the position of their choice was 0. Other low-scoring items were allowing a birth companion in the labor room, supporting to breastfeed within the first hour of delivery, answering in a clear, polite, and truthful manner, taking verbal consent before any action or procedure, providing information regarding contraception, and providing information about immunization of baby. The overall mean score for the non-consented care domain was 0.95.

**Table 3:** Domain of non-consented care. *: Reverse-coded items.

Sr. No.	Non-consented Care	Always/Yes	Sometimes	Never/No	Mean Score
		No. (%)	No. (%)	No. (%)	Mean (SD)
1	Did the service providers introduce themselves to you?	0 (0)	0 (0)	150 (100)	0 (0)
2	Did they allow the birth companion of your choice to accompany you?				
	a. In ward? (n=150)	128 (85.33)	0 (0)	22 (14.67)	1.71 (0.71)
	b. In labor room? (n=103)	01 (0.97)	0 (0)	102 (99.03)	0.01 (0.19)
3	Did they allow you and your birth companion to ask questions during examination, labor, and delivery?	46 (30.67)	77 (51.33)	27 (18.00)	1.13 (0.69)
4	Were your questions answered in a clear, polite, and truthful manner?	16 (10.67)	101 (67.33)	33 (22.00)	0.89 (0.56)
5	Did the staff take verbal consent before any action or practice, like major procedures, sampling, inserting an iv line, etc?	30 (20.00)	81 (54.00)	39 (26.00)	0.94 (0.67)
6	Did they allow you to deliver in your position of choice? (n=103)	0 (0)	0 (0)	103 (100)	0 (0)
7	Did the health providers explain how to push during a contraction and relax when the contraction disappears? (n=103) (47: LSCS)	80 (77.67)	0 (0)	23 (22.33)	1.55 (0.84)
8.*	Did they separate you from your baby without your consent? (n=128) (22: Neonatal intensive care unit (NICU) admission)	0 (0)	0 (0)	128 (100)	2 (0)
9	Did they support you in breastfeeding your baby within the first hour of delivery? (n=128)	44 (34.37)	0 (0)	84 (65.63)	0.68 (0.95)
10	Did they provide you with information about breastfeeding?	101 (67.33)	0 (0)	49 (32.67)	1.35 (0.93)
11	Did they provide you with information regarding contraception?	90 (60.00)	0 (0)	60 (40.00)	1.19 (0.99)

*Non-Confidential Care* 

**Figure 1:**
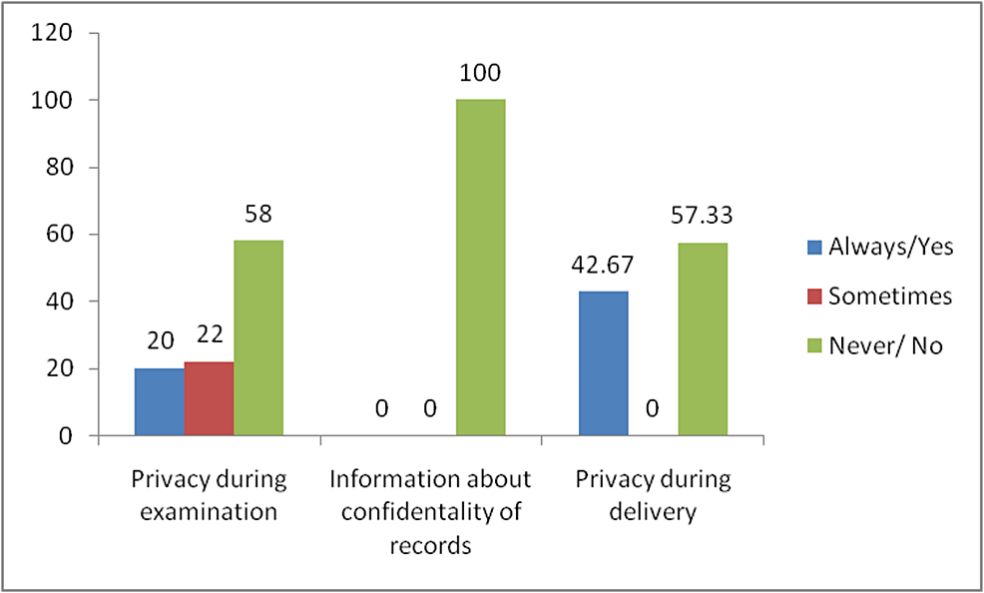
Non-confidential care in study subjects.

Non-confidential care in the study subjects is seen in Figure 1. Eighty-seven (58%) respondents reported that privacy was never maintained while carrying out a medical examination. None of the participants were assured that their recorded information would be kept safe and confidential. Also, 86 (57.33%) study subjects reported that their privacy was not maintained during delivery. So in this domain, items related to information regarding keeping the records safe and confidential had a mean score of 0. In contrast, maintenance of privacy during medical examination had a mean score of 0.62, and privacy during delivery had a mean score of 1.15. The overall mean score for the non-confidential care domain was 0.59.

Non-Dignified Care

Non-dignified care (including verbal abuse) in study subjects is shown in Table 4. About 94 (63%) females were greeted at least sometimes; however, 90 (60%) reported that the staff screamed or shouted at them sometimes or the other. Almost all females, 149 (99.33%), mentioned that the delivery table was cleaned before delivery. Also, 104 (69.33%) females felt the wards and the general environment of the health facility was always clean. However, only 54 (36%) females felt the toilets were clean and hygienic with working taps, flushes, and disposal systems. Thus, in the domain of non-dignified care, the items with lower mean scores were staff never greeting the study subjects, unhygienic and unclean toilets, overcrowded labor rooms and postnatal wards, and verbal abuse by staff. The overall mean score for the non-dignified care domain was 1.49.

**Table 4:** Non-dignified care in study subjects. *: Reverse-coded items.

Sr. No.	Non-Dignified Care	Always/Yes	Sometimes	Never/No	Mean Score
		No. (%)	No. (%)	No. (%)	Mean (SD)
1	Did the staff greet you?	03 (2)	91 (60.67)	56 (37.33)	0.64 (0.51)
2*	Did the staff scream or shout at you?	2 (1.33)	88 (58.67)	60 (40)	1.38 (0.51)
3	Was the labor table cleaned before your delivery?	149 (99.33)	0 (0)	01(0.67)	1.99 (0.08)
4*	Did you feel the health facility's labor rooms and postnatal wards were overcrowded?	0 (0)	101(67.33)	49 (32.67)	1.33 (0.47)
5	Did you feel the wards and the general environment of the health facility were clean?	104 (69.33)	46 (30.67)	0 (0)	1.69 (0.46)
6	Were the toilets clean and hygienic with working taps, flushes, and disposal systems?	54 (36)	92 (61.33)	4 (2.67)	1.33 (0.53)
7*	Were you threatened of disastrous consequences for you and your baby if the instructions of the health care providers were not followed? (n=103)	24 (23.3)	0 (0)	79 (76.70)	1.53 (0.85)
8*	Did you ever feel the health care providers were talking in their regional languages and making fun of you?	0 (0)	0 (0)	150 (100)	2 (0)

Discrimination Based on Specific Attributes

As far as the domain of discrimination based on specific attributes in the study subjects is concerned, none of them reported any discrimination or unequal treatment based on caste, religion, socioeconomic class, HIV/ HBsAg status, or disability. All the females mentioned that the staff spoke to them in a language they easily understood. The mean score of both the items was 2, which is the maximum score that can be attained.

Abandonment or Denial of Care

Abandonment or denial of care in study subjects is depicted in Table 5. Seventy-eight (52%) females mentioned that the staff responded quickly and efficiently to their needs and 99 (68%) of the females reported that they were provided periodic updates on the status and progress of labor at least sometimes. Also, 146 (97.33%) females reported that they were never left alone and unattended. In this domain, items having a mean score on the lower side were support in giving early skin-to-skin contact, encouraging to call for help if needed, explaining about immunization of newborn, providing periodic updates on the status and progress of labor, and responding quickly in case any help and information was needed. The overall mean score for abandonment or denial of care domain was 1.21.

**Table 5:** Domain of abandonment or denial of care. *: Reverse-coded items.

Sr. No.	Abandonment or denial of care	Always/Yes	Sometimes	Never/No	Mean Score
		No. (%)	No. (%)	No. (%)	Mean (SD)
1	Did they encourage you to call for help if you were in need?	24 (16)	31 (20.67)	95 (63.33)	0.52 (0.75)
2	Did the staff quickly respond to your need in case any help was required?	78 (52)	66 (44)	6 (4)	1.48 (0.57)
3	Did they provide periodic updates on the status and progress of labor to you? (n=147, 3: Placenta previa)	5 (3.40)	94 (63.95)	48 (32.65)	1.28 (0.52)
4*	Were you left alone and unattended?	1 (0.67)	3 (2)	146 (97.33)	1.97 (0.21)
5	Did they allow you and your baby to remain together at all times? (n=128, NICU=22)	128 (100)	0 (0)	0 (0)	2 (0)
6	Were you supported in giving early skin-to-skin contact to your baby? (n=128, NICU=22)	13 (10.16)	0 (0)	115 (89.84)	0.20 (0.61)
7	a. Was the treatment after discharge explained to you properly?	113 (75.33)	0 (0)	37 (24.67)	1.51 (0.87)
	b. Was the immunization of newborn explained to you properly?	53 (35.33)	0 (0)	97 (64.67)	0.71 (0.96)

Detention in the Facility

Finally, in the domain of detention in the facility, all the females, 150 (100%) responded that they were never detained in the hospital for unjustified reasons. However, about 97 (65%) females reported that there were demands for informal payments/bribes, and thus, this was the lower scoring item in this domain with a mean score of 0.7 (SD: 0.95). The overall mean score for the domain of detention in the facility was 1.35.

**Figure 2:**
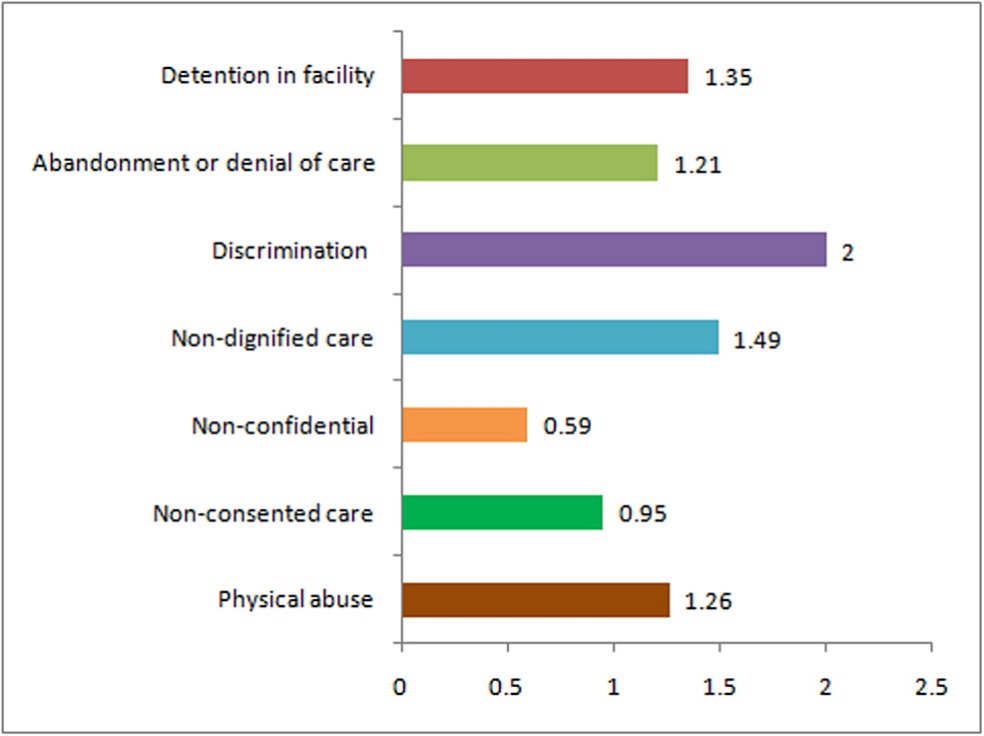
Mean score of various domains of respectful maternity care.

All the women in the present study experienced at least one form of disrespect at the hospital during labor, childbirth, or postnatal period. A bar diagram showing the mean score for each domain of respectful maternity care is seen in Figure 2. The lowest scoring domain was non-confidential care (0.59), followed by non-consented (0.95), abandonment or denial of care (1.21), and physical abuse (1.26). The top-scoring domain was discrimination based on specific attributes with the highest possible score of 2. However, for all other domains, the score was less than 1.5, which is a cause for concern and requires concentrated efforts to make the care more respectful. Special care is required for non-confidential and non-consented care domains, for which the mean score was less than 1.

## Discussion

Over the past few decades, the concept of respectful maternity care has evolved and expanded to include diverse perspectives and frameworks. The present cross-sectional study was conducted to assess the current status of respectful maternity care in a tertiary care center in central India. In this study, all the women experienced at least one form of disrespect during their labor, childbirth, or postnatal period at the health facility, which is an alarming sign that highlights the importance of making maternity care more respectful and abuse-free. The results of our study are similar to that of the studies conducted by Singh A et al. [10] and Okafor II et al. [11]. A higher proportion of disrespect and abuse was also reported by other studies [12-15]. However, the proportion of disrespect and abuse reported by Goli S et al. [16] and Pathak P and Ghimire B [17] was much lower than that observed in our study.

As per the findings of the present study, approximately half of the respondents reported the use of physical force or abusive behavior by health care providers in some form, including pushing, slapping, pinching, or any gesture towards slapping or hitting during examination and delivery. However, this figure appears to be on the higher side. It can be explained by the fact that, at times, situations force the health care providers to resort to raised voices and physically pressure the women to bear down for the safety and wellbeing of both mother and child. Findings similar to our study were also reported by Okafor II et al. [11] and Asefa A et al. [18]. However, the reported proportion of physical abuse was lesser in some other studies [13, 17, 19, 20].

Introducing oneself helps the health care providers to build a rapport with the mothers and aids in alleviating their anxiety. However, in the present study, all the females reported that the service providers never introduced themselves. Our findings are consistent with the study done by Afulani PA et al. [21], wherein 90% of women reported that none of the providers introduced themselves. The findings of the present study can be well explained by the fact that it was carried out in a public health facility wherein a dearth of human resources and overcrowded wards make it difficult for the health care providers to greet every woman and introduce themselves. Birth companions were not allowed to accompany most females in labor rooms in our study. Similar findings were also noted by Sethi R et al. [22] and Singh A et al. [10]. The study setting being a public sector institute, accompaniment of a birth companion could have resulted in unnecessary overcrowding and additional chances of infections, adversely impacting patient care. Birth companions are known to provide continuous labor support for women contributing to positive birth outcomes and satisfaction. Hence recommended practice of birth companionship is integral to respectful maternity care throughout labor and childbirth.

As reported by most studies, non-consented care was one of the most common forms of disrespect in our study, too [11, 13, 14, 18, 20]. This occurrence is also explained by overcrowded wards, which make obtaining consent for minor non-invasive procedures much more difficult. However, a heartening finding that emerged from this study was that none of the service providers ever separated any of the mothers from their babies without their consent. 

In our study, more than half of the respondents reported that privacy was never maintained during medical examination and delivery. Probably infrastructural constraints and scarcity of resources in a public health setup make it difficult to maintain adequate privacy. Akin to the present study findings, Manu A et al. [23] and Sethi R et al. [22] reported that privacy was not ensured for women during delivery. However, violation of privacy in relatively fewer study participants was reported by other authors [14, 18, 24].

One-third of the females expressed that the health care providers never greeted them, and almost two-thirds of the participants reported that the staff screamed or shouted at them at least once. Verbal abuse was also reported in studies carried out across the globe [17, 21, 22, 24]. The findings of our study can be explained by the fact that the focus of the public health sector is mainly on the provision of maternity care to a large chunk of the population. While doing so somewhere, the respectful component knowingly or unknowingly gets compromised. 

A promising feature noted in this study was that none of the study subjects was ever discriminated against based on any specific attribute. All the females mentioned that the staff spoke to them in a language they easily understood. This encouraging finding will accentuate the general population's trust in the public sector healthcare system in the long run. Similar to the findings of our study, a lesser proportion of discrimination was reported in other study settings [13, 17, 20, 24].

Most of the females in this study reported that they were never encouraged to call for help when in need and that the staff did not respond quickly, which is consistent with the findings of other studies [17, 25,26]. This untoward occurrence might be due to the over-exhaustion of the nursing staff, as the recommended staff-to-patient ratio is difficult to maintain in a public setup.

Almost all of the females in our study reported that they were never left alone and unattended, which is a reassuring finding. Periodic updates on the status and progress of labor were also provided to almost two-thirds of the respondents, which was consistent with the findings of Asefa A et al. [15]. All the females responded that they were never detained in the hospital for unjustified reasons. Findings similar to our study are reported by other authors [15, 24]. However, detention against the will of the respondents was reported by many other authors [11, 13, 18-20]. About 65% of the females in our study reported that there were demands for informal payments/bribes/bakshish. Similarly, a high proportion of inappropriate demands for money were reported by Bhattacharya S et al. [19] and Rajkumari B et al. [12], while this figure was considerably low in a study carried out by Baranowska B et al. [14]. This is because celebrating childbirth is universal in Indian culture, and sometimes the relatives offer sweets and 'bakshish' of their own will to mark the joyous occasion. The domains requiring concentrated efforts to rectify and make the care more respectful are non-confidential care, non-consented care, abandonment or denial of care, and physical abuse. However, the absence of discrimination based on specific attributes points towards equitable care, a positive feature of a public institute. 

Strengths and limitations of this study

An essential strength of this study is that the postpartum mothers were interviewed within the first three to five days post-delivery. This time frame significantly reduces the recall bias. However, the current study also has a few limitations inherent to the study design's cross-sectional nature. Also, generalizability may be limited because this is a hospital-based study conducted in a single institute with a non-random sampling method. All occurrences of mistreatment are self-reported, which could have led to either over or under-reporting. Additionally, in the study context, not reporting unpleasant facts associated with delivery (an event that is already over) could have led to 'courtesy bias.'

Recommendations

Depending on the feasibility, the constitution of quality control teams comprising doctors, nurses, and sanitary supervisors is recommended to optimize the quality of respectful maternity care. Proper implementation of respectful maternity care includes learning new and innovative techniques and unlearning some traditionally followed ones. Hence it is recommended that health care providers of all cadres should be trained and retrained periodically to inculcate attitudes and practices that would make maternity care more respectful. Furthermore, it is recommended that multicentric studies with larger sample sizes and different study designs be carried out. Also, mixed-methods studies are proposed to understand the complex and interactive patient-provider context of disrespect and abuse, which may provide ways of reducing and eliminating the same. Evidence from such studies will provide essential guidance to policymakers for effective implementation.

## Conclusions

The findings of this study provide a broad array of insights into the present status of respectful maternity care in the study setting. With the increasing number of deliveries occurring in the hospital, challenges in providing respectful maternity care persist, with mothers continuing to be disrespected and abused during childbirth. Almost all of the respondents faced some form of disrespect in one domain or the other. However, non-confidential and non-consented care domains were the lowest scoring ones. This calls for concentrated efforts in these areas, ultimately making the care more dignified and respectful. It is evident from the findings of the present study that although mothers are noticing and reporting positive changes in maternity care practices, respectful maternity care still has a long way to go.

With the majority of the Indian studies primarily focusing on the availability and accessibility of maternal healthcare services, the area of respectful maternity care remains largely unexplored, leaving a research gap. Hence the present study serves as a benchmark for filling this vital research gap with robust evidence of disrespect and abuse experienced by women availing maternity services in healthcare facilities. 
